# Systematic Review of Cultural Expressions of Depression in African Communities; Implications for Service Provision

**DOI:** 10.1192/bjo.2024.224

**Published:** 2024-08-01

**Authors:** Victoria Ozidu, Hani Dourado

**Affiliations:** Greater Manchester Mental Health Trust, Manchester, United Kingdom

## Abstract

**Aims:**

This review delves into the understanding of depression within African communities, extending its scope to nations with significant African populations, aiming to enhance service provision for these patients. While focusing on cultural experiences of depression that transcend geographical boundaries, it builds upon existing literature predominantly centred on sub-Saharan African countries.

**Methods:**

A comprehensive literature search was conducted across multiple databases, yielding 13 relevant articles after applying stringent criteria. Following Cochrane guidelines, search terms encompassed population (Africa, Africans, African communities), exposure (Depression, Depressive disorder, Dysphoria, Dysthymia, Low mood), and outcomes (Cultural expressions, Cultural variations, Somatization, Cultural framework, Cross-cultural research, Service provision).

**Results:**

Analysing selected articles through the CASP checklist, a narrative synthesis of qualitative studies over the past twelve years elucidated diverse perceptions and expressions of depression in African communities compared with Western contexts. Three major themes emerged: Expressions of depression (with subthemes: Attitudes towards depression), Perceptions of depression (including Stigmatization), and culturally acceptable forms of treatment (including Barriers towards treatment).

**Conclusion:**

The review underscores the significance of integrating culturally acceptable treatment methods into psychological therapy for improved healthcare delivery. Collaboration between clinicians and patients is pivotal, with religious assistance emerging as a culturally acceptable treatment avenue. Establishing therapeutic alliances with religious communities could enhance treatment effectiveness. Further research is warranted to explore the impact of religious activity on depression symptoms and progression, as well as the influence of mental health providers' religious backgrounds on treatment dynamics. This holistic approach is crucial for addressing the unique cultural nuances surrounding depression in African communities and optimizing patient care.

